# Phylogenetic Resolution in *Juglans* Based on Complete Chloroplast Genomes and Nuclear DNA Sequences

**DOI:** 10.3389/fpls.2017.01148

**Published:** 2017-06-30

**Authors:** Wenpan Dong, Chao Xu, Wenqing Li, Xiaoman Xie, Yizeng Lu, Yanlei Liu, Xiaobai Jin, Zhili Suo

**Affiliations:** ^1^State Key Laboratory of Systematic and Evolutionary Botany, Institute of Botany, Chinese Academy of SciencesBeijing, China; ^2^Peking-Tsinghua Center for Life Sciences, Academy for Advanced Interdisciplinary Studies, Peking UniversityBeijing, China; ^3^University of Chinese Academy of SciencesBeijing, China; ^4^Shandong Provincial Center of Forest Tree Germplasm ResourcesJinan, China; ^5^Beijing Botanical Garden, Institute of Botany, Chinese Academy of SciencesBeijing, China

**Keywords:** *Juglans*, complete chloroplast genome, small inversion, internal transcribed spacer, ubiquitin ligase gene, phylogeny

## Abstract

Walnuts (*Juglans* of the Juglandaceae) are well-known economically important resource plants for the edible nuts, high-quality wood, and medicinal use, with a distribution from tropical to temperate zones and from Asia to Europe and Americas. There are about 21 species in *Juglans*. Classification of *Juglans* at section level is problematic, because the phylogenetic position of *Juglans cinerea* is disputable. Lacking morphological and DNA markers severely inhibited the development of related researches. In this study, the complete chloroplast genomes and two nuclear DNA regions (the internal transcribed spacer and ubiquitin ligase gene) of 10 representative taxa of *Juglans* were used for comparative genomic analyses in order to deepen the understanding on the application value of genetic information for inferring the phylogenetic relationship of the genus. The *Juglans* chloroplast genomes possessed the typical quadripartite structure of angiosperms, consisting of a pair of inverted repeat regions separated by a large single-copy region and a small single-copy region. All the 10 chloroplast genomes possessed 112 unique genes arranged in the same order, including 78 protein-coding, 30 tRNA, and 4 rRNA genes. A combined sequence data set from two nuclear DNA regions revealed that *Juglans* plants could be classified into three branches: (1) section *Juglans*, (2) section *Cardiocaryon* including *J. cinerea* which is closer to *J. mandshurica*, and (3) section *Rhysocaryon*. However, three branches with a different phylogenetic topology were recognized in *Juglans* using the complete chloroplast genome sequences: (1) section *Juglans*, (2) section *Cardiocaryon*, and (3) section *Rhysocaryon* plus *J. cinerea*. The molecular taxonomy of *Juglans* is almost compatible to the morphological taxonomy except *J. cinerea* (section *Trachycaryon*). Based on the complete chloroplast genome sequence data, the divergence time between section *Juglans* and section *Cardiocaryon* was 44.77 Mya, while section *Rhysocaryon* diverged from other sections in the genus *Juglans* was 47.61 Mya. Eleven of the 12 small inversions in the chloroplast genomes provided valuable phylogenetic information for classification of walnut plants at section and species levels. Our results are valuable for future studies on *Juglans* genetic diversity and will enhance the understanding on the phylogenetic evolution of Juglandaceae.

## Introduction

Walnuts (*Juglans* L.) are well-known economically important resource trees for the edible nuts, high-quality wood, and medicinal use. *Juglans*, one of the eight living genera in the family Juglandaceae, has about 21 species in the world, with a distribution from tropical to temperate zones, and from Asia to Europe and Americas (Lu, 1982; Lu et al., 1999; APG III, 2009; Pei and Lu, 2011; Suo et al., 2012a; APG IV, 2016).

*Juglans* plants were classified into four sections according to morphology of leaf, floral, and fruit characteristics, i.e., section *Rhysocaryon*, section *Cardiocaryon*, section *Trachycaryon*, and section *Juglans* (syn. section *Dioscaryon*) (Dode, 1909; Manning, 1978; Lu et al., 1999). Manning (1978) and McGranahan and Leslie (1991) presented complete descriptions of the morphological variation, ecological distribution, and taxonomic treatment of the genus *Juglans*. Manchester (1987) considered that plants of the genus *Juglans* could be classified into three sections, and merged section *Trachycaryon* (*Juglans cinerea*) and section *Cardiocaryon* together according to the consistency of the walnut fossil characteristics.

*Juglans* section *Juglans* includes the two cultivated walnuts, *J. regia* with a distribution from southeastern Europe to China and the Himalayas, and *J. sigillata* distributed in Yunnan, Guizhou, Sichuan and Tibet of Southwest China. The nut of section *Juglans* is distinguished from that of related species by a dehiscent husk thin shell at fruit mature stage and narrow septum separating the kernel halves, all of which greatly facilitate kernel extraction (Dode, 1909; Manning, 1978; Lu et al., 1999; Martinez-Garcia et al., 2016). More than 300 walnut cultivars are documented under *J. regia* for producing edible nuts. *J. regia* has been cultivated for more than 6800 years (Beer et al., 2008; Joly and Visset, 2009; Pei and Lu, 2011). Section *Trachycaryon*, native to eastern North America, comprises a single species, *J. cinerea* L.. Section *Rhysocaryon*, endemic to North and South Americas (Stone et al., 2009; Suo et al., 2012a), consists of 16 taxa: *J. australis*, *J. boliviana*, *J. californica*, *J. guatemalensis*, *J. jamaicensis*, *J. hindsii*, *J. hirsute*, *J. major*, *J. microcarpa*, *J. mollis*, *J. neotropica*, *J. nigra*, *J. olanchana*, *J. pyriformis*, *J. steyermarkii*, and *J. venezuelensis*. Section *Cardiocaryon* contains three taxa native to East Asia: *J. manshurica*, *J. cathayensis*, and *J. ailantifolia*.

Molecular biological studies supported the sectional classification of *Juglans* based on morphological characteristics, except for section *Trachycaryon* represented by the single species *J. cinerea. J. cinerea* was placed within section *Cardiocaryon* when analysis was conducted using nuclear DNA sequence (the internal transcribed spacer, ITS), but within section *Rhysocaryon* when analysis was conducted using cpDNA sequences (NCS and *matK*) (Stanford et al., 2000; Aradhya et al., 2007). The phylogenetic position of *J. cinerea* is thus still problematic. Haplotype phylogeography suggested a geographical differentiation prior to the last glacial advance in eastern populations and separate postglacial migration paths for eastern and western populations when the detection was conducted using sequences from eight chloroplast DNA regions (Laricchia et al., 2015).

As a matter of fact, the plasticity of morphological traits is frequently observed due to influences from environmental conditions and different developmental stages. The internal transcribed spacer (ITS) sequence of nuclear ribosomal DNA and cpDNA fragments (*rbcL*, *matK*, and *trnH–psbA*) commonly recommended to use have only limited resolution in identifying closely related taxa in the Juglandaceae (Xiang et al., 2011; Dong et al., 2014, 2015; Suo et al., 2015). Thus, the phylogenetic relationship at section level in *Juglans* is still a challenging task, because of lacking morphological and DNA markers (Gunter et al., 1994; Cosmulescu and Botu, 2012; Ciarmiello et al., 2013; Suo et al., 2015). It is necessary to explore more genetic information for phylogenetic reconstruction of *Juglans*.

In recent years, the chloroplast genomes have been proven successfully to be more informative than cpDNA fragments in revealing phylogeny of land plants (Jansen et al., 2007; He et al., 2012; Suo et al., 2012b; Dong et al., 2016; Xu et al., 2017). Hu et al. (2017) used the complete chloroplast genome information to discuss genetic divergence of five Chinese *Juglans* taxa in comparison with the Fagaceae and the Betulaceae, the families closely related to the Juglandaceae. New nuclear DNA markers from the ubiquitin–proteasome system related DNA regions showed higher sensitivity and better resolution in detecting genetic diversity in genera *Juglans* and *Lagerstroemia* (Suo et al., 2015, 2016). The ubiquitin–proteasome system, which plays a key role in degradation of proteins, is imperative for maintaining the cellular homeostasis in eukaryotic cells (Ganoth et al., 2013; Marin, 2013). Furthermore, it has been reported that micro-structure mutations, such as small inversions, in chloroplast genomes may have a potential application value in the phylogenetic analysis of land plants (Kelchner and Wendel, 1996; Kim and Lee, 2005; Borsch and Quandt, 2009; Morrison, 2009).

The small size inversions (∼50 bp) are probably to be generated by intra-molecular recombination events (Ogihara et al., 1988; Hiratsuka et al., 1989). The possession of the same inversion is regarded as reliable evidence of shared ancestry (Jansen and Palmer, 1987; Doyle et al., 1992, 1996; Kim and Lee, 2004). The inverted repeats formed the stem structures and the small inversions formed the loops.

In this study, we report nine newly sequenced complete chloroplast genomes from *Juglans* (eight species and one cultivar). In addition, sequences from two nuclear DNA regions (ITS, and ubiquitin ligase gene), were also used to help resolving the genetic diversity in *Juglans*. The aims of our study are: (1) to upgrade the understanding on the application value in phylogenetic resolution of *Juglans*, (2) to provide more genetic resources for obtaining a better resolution on the phylogeny of the genus *Juglans*, and (3) to deepen the understanding on the genetic and evolutionary significance from the structural diversity of the chloroplast genomes.

## Materials and Methods

### Plant Materials and DNA Extraction

Fresh leaves were collected from the trees of *J. nigra*, *J. major*, and *J. regia* ‘Bokexiang’ growing in the Resources Nursery of the Forestry Bureau of Luoning County, Henan Province, China; *J. sigillata*, *J. cathayensis*, and *J. hindsii* from the Arboretum of the Forestry Academy of Yunnan Province, Kunming City, Yunnan Province, China; *J. mandshurica* growing in the Beijing Botanical Garden of the Chinese Academy of Sciences. *J. regia* from the plant of a natural population located in Taihang mountainous region of Yixian County, Hebei Province, China, and dried leaves of *J. cinerea* were taken from voucher specimen, 01816245, Chinese National Herbarium, collected May 5, 2006, at Sevier County, Tennessee, United States, No. 2274609 from PE Herbarium (**Table 1**). The (fresh) leaves from each accession were immediately dried with silica gel for further DNA extraction. Total genomic DNAs were extracted from each sample using the Plant Genomic DNA Kit (DP305) from Tiangen Biotech (Beijing) Co., Ltd., China.

**Table 1:** Taxa of *Juglans* used in this study.

				GenBank accession numbers	
				
No.	Taxon	Section	Place of collection	Chloroplast genome	ITS/UBL
1	*J. regia*	*Juglans*	Taihang mountainous region in Yixian County, Hebei Province, China	MF167464	MF182370/MF279072
2	*J. regia* ‘Bokexiang’	*Juglans*	Resources Nursery, Forestry Bureau of Luoning County, Henan Province, China	MF167463	MF182375/MF279073
3	*J. sigillata*	*Juglans*	Arboretum, Forestry Academy of Yunnan Province, Kunming City, Yunnan Province, China	MF167465	MF182371/KF994009
4	*J. cathayensis*	*Cardiocaryon*	Arboretum, Forestry Academy of Yunnan Province, Kunming City, Yunnan Province, China	MF167457	MF182373/MF279074
5	*J. mandshurica*	*Cardiocaryon*	Beijing Botanical Garden of the Chinese Academy of Sciences, China	MF167461	MF182374/KF994012
6	*J. hindsii*	*Rhysocaryon*	Arboretum, Forestry Academy of Yunnan Province, Kunming City, Yunnan Province, China	MF167459	MF182369/KF589931
7	*J. major*	*Rhysocaryon*	Resources Nursery, Forestry Bureau of Luoning County, Henan Province, China	MF167460	MF182376/KF589930
8	*J. nigra*	*Rhysocaryon*	Resources Nursery, Forestry Bureau of Luoning County, Henan Province, China	MF167462	MF182372/KF589927
9	*J. cinerea*	*Trachycaryon*	Voucher specimen (No. 2274609) from Sevier County of Tennessee, United States in Herbarium of Institute of Botany, Chinese Academy of Sciences, Beijing, China	MF167458	MF182366/MF182377


**ITS, internal transcribed spacer, partial sequence; UBL, ubiquitin ligase gene partial sequence.**

### Chloroplast Genome Sequencing and Assembling

Four *Juglans* chloroplast genomes of *J. regia*, *J. regia* ‘Bokexiang’, *J. sigillata* and *J. mandshurica* were sequenced using the short-range PCR (Polymerase Chain Reaction) method reported by Dong et al. (2012, 2013). The PCR protocol was as follows: preheating at 94°C for 4.5 min, 34 cycles at 94°C for 50 s, annealing at 55°C for 40 s, and elongation at 72°C for 1.5 min, followed by a final extension at 72°C for 8 min. PCR amplification was performed in an Applied Biosystems VeritiTM 96-Well Thermal Cycler (Model#: 9902, made in Singapore). The amplified DNA fragments were sent to Shanghai Majorbio Bio-Pharm Technology Co., Ltd. (Beijing) for Sanger sequencing in both the forward and reverse directions using a 3730xl DNA analyzer (Applied Biosystems, Foster City, CA, United States). The chloroplast DNA sequences were manually confirmed and assembled using Sequencher (v5.4) software.

*Juglans cathayensis*, *J. cinerea*, *J. hindsii*, *J. major*, and *J. nigra* (Supplementary Table S1) were sequenced using Illumina HiSeq 4000. Before sequencing, paired-end libraries with 300-bp insert size were constructed following the manufacturer’s protocol (Illumina Inc.). 303,763–1,744,889 mapped reads were obtained from 8,801,265–29,818,482 raw reads (Supplementary Table S1). The length of sequencing reads was 150 bp. The four junctions between the inverted repeat region (IRs) and the small single copy (SSC)/large single copy (LSC) region were checked by amplification using specific primers, followed by Sanger sequencing (Dong et al., 2013).

The high-throughput sequencing data were qualitatively assessed and assembled using SPAdes 3.6.1 (Bankevich et al., 2012). Using *J. regia* (KT963008) as a reference sequence, we selected chloroplast genome contigs using Blast method. The contigs of the chloroplast genome were assembled using Sequencher (v5.4) with default parameters and the gaps between contigs were filled in by amplification with PCR-based conventional Sanger sequencing using ABI 3730. The specific primers were designed based on the flanking sequences to bridge the gaps. After that, all reads were mapped to the spliced chloroplast genome sequence using Geneious 8.1 (Kearse et al., 2012) to avoid assembly errors.

### Genome Annotation

Chloroplast genome annotation was performed using the Dual Organellar Genome Annotator (DOGMA) (Wyman et al., 2004). BLASTX and BLASTN searches were employed to accurately annotate the protein-encoding genes and to identify the locations of the ribosomal RNA (rRNA) and transfer RNA (tRNA) genes. Gene annotation information from other closely related plant species was also utilized for sure when the boundaries of the exons or introns could not be precisely determined because of the limited power of BLAST in chloroplast genome annotation. The chloroplast genome map was drawn using Genome Vx software (Conant and Wolfe, 2008). The nine chloroplast genomes newly sequenced in this study were deposited in GenBank (accession numbers MF167457-MF167465).

### PCR Amplification of the Two Nuclear DNA Regions

The ITS sequences were amplified using the primer pair, ITS-u1 and ITS-u4, and following the PCR amplification conditions as reported by Cheng et al. (2016). The DNA sequence from the ubiquitin ligase gene region (UBE3) was amplified using the primer pair, H_UBE3_23f and H_UBE3_838r, and following the PCR amplification conditions as reported by Suo et al. (2015). The eleven ITS sequences and one ubiquitin ligase gene sequence were deposited in GenBank (accession numbers MF182366-MF182377). The ubiquitin ligase gene sequences of other samples used for comparative analysis in this study were downloaded from GenBank (accession numbers: KF994007-KF994018) (Suo et al., 2015). The DNA sequences of outgroups were also deposited in GenBank (*Pterocarya stenoptera*, MF182367 for ITS, KF994018 for UBE3; *Cyclocarya paliurus*, MF182368 for ITS, KF994017 for UBE3).

### Sequence Divergence Analysis

The chloroplast genome sequences were aligned using MAFFT (Katoh and Standley, 2013) and were manually adjusted using Se-Al 2.0 (Rambaut, 1996). Variable and parsimony-informative base sites across the complete chloroplast genomes, the large single copy (LSC), small single copy (SSC), and inverted repeat (IR) regions of the chloroplast genomes were calculated using MEGA 6.0 software (Tamura et al., 2013). Sliding window analysis was conducted to generate nucleotide diversity (Pi) of the chloroplast genome using DnaSP (DNA Sequences Polymorphism version 5.10.01) software (Librado and Rozas, 2009). The step size was set to 200 bp, with a 600-bp window length. Repeating sequences were scanned over the complete chloroplast DNA sequences, species by species, using the REPuter program. Probable inversion regions associated with the repeated sequences were evaluated by detailed alignment and sequence similarity searches (Kurtz et al., 2001; Kim and Lee, 2005).

### Phylogenetic Analysis

Maximum parsimony (MP) analyses were performed using PAUP v4b10 (Swofford, 2003). All characters were equally weighted, gaps were treated as missing, and character states were treated as unordered. Heuristic search was performed with MULPARS option, tree bisection-reconnection (TBR) branch swapping, and random stepwise addition with 1,000 replications. The Maximum likelihood (ML) analyses were conducted using RAxML 8.0 (Stamatakis, 2006). For ML analyses, the best-fit model, general time reversible (GTR)+G was used in all analysis as suggested with 1,000 bootstrap replicates.

Bayesian inference (BI) was conducted with Mrbayes v3.2 (Ronquist et al., 2012). The Markov chain Monte Carlo (MCMC) analysis was run for 2 × 5,000,000 generations. Trees were sampled at every 1,000 generations with the first 25% discarded as burn-in. The remaining trees were used to build a 50% majority-rule consensus tree. The stationarity was regarded to be reached when the average standard deviation of split frequencies remained below 0.01.

### Estimation of Divergence Times

The BEAST v2.3.3 package (Bouckaert et al., 2014) was used to analyze the chloroplast genome dataset for assessment of *Juglans* divergence times using a relaxed molecular clock method (Drummond et al., 2006). We selected chloroplast genome dataset for divergence time analysis. For calibration, two constraints were used: (1) The age for the most recent common ancestor of the Juglandaceae was set to 79.9 Mya (71.2–96.4) and assigned a normal distribution (Xiang et al., 2014); (2) the *Juglans* crown group was set to age of 45 Mya (Manchester, 1987; Aradhya et al., 2007). We used an uncorrelated log-normal clock, a Yule tree prior, and a randomly generated starting tree. The data was assigned a GTR + I + G model of substitution. Runs were conducted for 500 million generations with parameters sampled every 5,000 steps. Tracer v.1.6 (Rambaut et al., 2014) was used to check convergence and stationarity, to determine the number of generations discarded as burn-in, and to confirm that effective sample size (ESS) values were over 200.

## Results

### Chloroplast Genome Features

**FIGURE 1:**
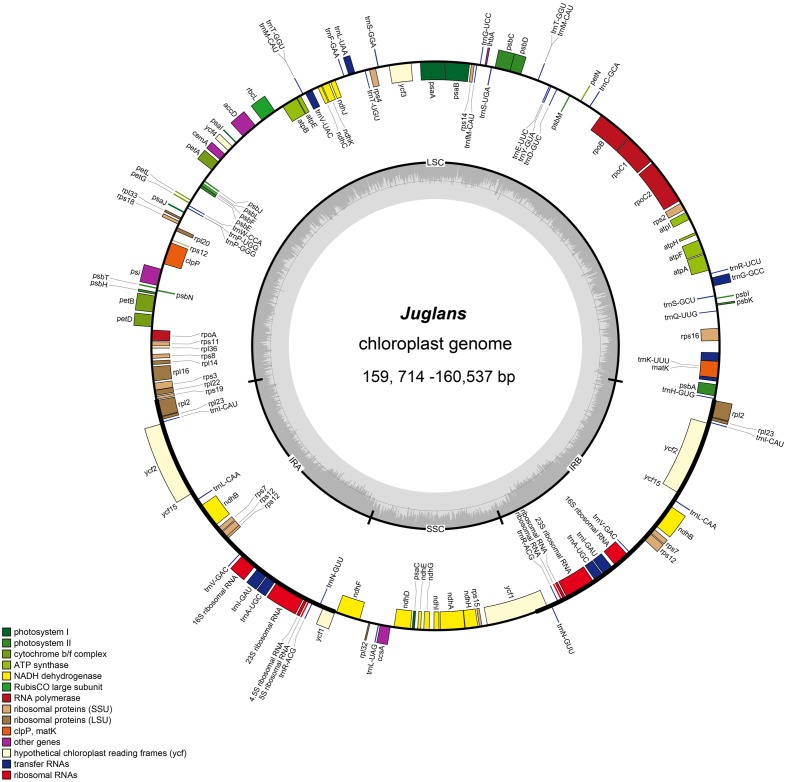
Gene map of *Juglans* chloroplast genome. The genes inside and outside of the circle are transcribed in the clockwise and counterclockwise directions, respectively. Genes belonging to different functional groups are shown in different colors. The thick lines indicate the extent of the inverted repeats (IRa and IRb) that separate the genomes into small single-copy (SSC) and large single-copy (LSC) regions.

The *Juglans* complete chloroplast genomes ranged from 159,714 (*J. hopeiensis*, GenBank accession no. KX671977) to 160,537 base pairs (bp) (*J. regia* voucher JREG20151001, GenBank accession no. KT870116) in length. All the chloroplast genomes possessed the typical quadripartite structure of angiosperms, consisting of a pair of the inverted repeat region (IRs: 26,023–26,039 bp) separated by a large single-copy region (LSC: 89,307–89,917 bp) and a small single-copy region (SSC: 18,352–18,429 bp) (**Figure 1** and **Table 2**). All the 10 chloroplast genomes possessed 112 unique genes arranged in the same order, including 78 protein-coding, 30 tRNA, and 4 rRNA genes. GC content in each chloroplast genome is identically 36.1% (Supplementary Table S2).

**Table 2:** Summary of complete chloroplast genome features of the 10 *Juglans* taxa.

	Section			Section			Section			Section
				
	*Juglans*			*Cardiocaryon*			*Rhysocaryon*			*Trachycaryon*
				
Name of taxon	*J. regia*	‘Bokexiang’	*J. sigillata*	*J. hopeiensis*	*J. cathayensis*	*J. mandshurica*	*J. hindsii*	*J. major*	*J. nigra*	*J. cinerea*
Genome length	160,352	160,370	160,351	159,714	159,734	159,729	160,406	160,276	160,274	160,288
LSC length	89,871	89,873	89,871	89,316	89,314	89,307	89,917	89,829	89,807	89,803
IR length	26,034	26,034	26,034	26,023	26,023	26,023	26,032	26,025	26,039	26,034
SSC length	18,413	18,429	18,412	18,352	18,374	18,376	18,425	18,397	18,389	18,417
Total gene number	112	112	112	112	112	112	112	112	112	112
No. of protein coding genes	78	78	78	78	78	78	78	78	78	78
No. of rRNA genes	4	4	4	4	4	4	4	4	4	4
No. of tRNA genes	30	30	30	30	30	30	30	30	30	30
GC content in genome (%)	36.1	36.1	36.1	36.1	36.1	36.1	36.1	36.1	36.1	36.1


*>‘Bokexiang’ = *J. regia* ‘Bokexiang’.*

### Genome Sequence Divergence

The distribution of each single nucleotide polymorphic site (SNP) among the 10 *Juglans* chloroplast genomes is shown in Supplementary Table S3. There were 721 SNPs in LSC region, 268 in SSC region, and 30 in each of the IR regions.

At section level, the largest sequence divergence occurred between *Juglans* sections *Cardiocaryon* and *Rhysocaryon*, with the largest nucleotide substitution number (512 to 575) and the largest sequence distance (0.0032 to 0.0036). The smallest sequence divergence at section level was observed between sections *Juglans* and *Cardiocaryon*, with the lowest nucleotide substitution number (465 to 471) and the lowest sequence distance (0.0029 to 0.0030). The sequence divergence between sections *Juglans* and *Rhysocaryon* was observed to be intermediate, with the nucleotide substitution number ranged from 492 to 545, and the sequence distance ranged from 0.0031 to 0.0034 (**Table 3**).

**Table 3:** Numbers of the pairwise nucleotide substitutions (the lower triangle) and sequence distance (the upper triangle) between the 10 complete cp genomes representing four sections within genus *Juglans*.

Section	Name	*J. regia*	‘Bokexiang’	*J. sigillata*	*J. hopeiensis*	*J. cathayensis*	*J. mandshurica*	*J. hindsii*	*J. major*	*J. nigra*	*J. cinerea*
*Juglans*	*J. regia*		0	0	0.0030	0.0029	0.0029	0.0031	0.0034	0.0032	0.0034
	‘Bokexiang’	7		0	0.0029	0.0029	0.0029	0.0031	0.0033	0.0032	0.0034
	*J. sigillata*	4	7		0.0030	0.0030	0.0029	0.0031	0.0034	0.0032	0.0034
*Cardiocaryon*	*J. cathayensis*	469	467	470	0.0006		0.0006	0.0032	0.0035	0.0033	0.0036
	*J. hopeiensis*	470	468	471		93	0	0.0033	0.0035	0.0034	0.0036
	*J. mandshurica*	467	465	468	6	99		0.0032	0.0035	0.0033	0.0036
*Rhysocaryon*	*J. hindsii*	493	492	493	523	512	514		0.0011	0.0009	0.0011
	*J. major*	534	532	534	559	557	557	170		0.0011	0.0014
	*J. nigra*	512	509	512	537	528	528	143	182		0.0007
*Trachycaryon*	*J. cinerea*	545	542	545	575	566	566	175	219	113	


**‘Bokexiang’ = *J. regia* ‘Bokexiang’.**

The lowest within-section-divergence was observed in section *Juglans*, with the lowest nucleotide substitutions of 4 to 7, and the lowest sequence distance of zero. The largest within-section-divergence was found in section *Rhysocaryon*, with the largest nucleotide substitutions of 143 to 182, and the largest sequence distance of 0.0009 to 0.0011. The intermediate within-section-divergence was observed in section *Cardiocaryon* (**Table 3**).

At taxon level, the largest sequence divergence was observed between *J. cinerea* and *J. hopeiensis*, with the highest nucleotide substitution number (575) and the highest sequence distance (0.0036). The lowest divergence was between *J. cinerea* and *J. nigra* of section *Rhysocaryon*, with the lowest nucleotide substitution number (113) and the lowest sequence distance (0.0007) (**Table 3**). *J. hopeiensis* is classified in section *Cardiocaryon* and is closer to *J. mandshurica*.

**FIGURE 2:**
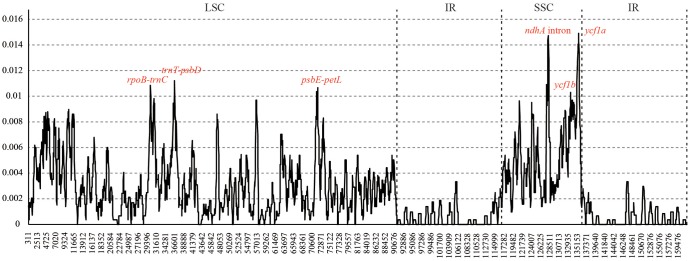
Sliding window analysis of the whole chloroplast genomes of 10 *Juglans* species. (window length: 600 bp, step size: 50 bp). *X*-axis: position of the midpoint of a window, *Y*-axis: nucleotide diversity of each window.

Six hyper-variable regions (Pi > 0.01) were uncovered among the sampled *Juglans* taxa. They are three intergenic spacers (*rpoB-trnC*, *trnT-psbD*, and *psbE-petL*) from the LSC region, and two gene regions (*ycf1b* and *ycf1a*) and one intron of *ndhA* from SSC region (**Figure 2**).

### Small Inversions

It has been reported that each small inversion is commonly associated with a hairpin secondary structure in the chloroplast genomes (Kim and Lee, 2005). In this study, a total of 12 small inversions were uncovered based on the sequence alignment of the 10 complete chloroplast genomes representing the four sections of the genus *Juglans*, of which nine small inversions were located in LSC region, two in IR region, and one in SSC region. Eleven of the 12 small inversions were seen in intergenic spacers, and one of them was in *ycf1* gene region of the chloroplast genomes (**Table 4**).

**Table 4:** The location and length of 12 small inversions.

					Direction of the small inversions in each taxon of the four sections									
					
			Length (bp)		Section *Juglans*			Section *Cardiocaryon*			Section *Rhysocaryon*			Section *Trachycaryon*
							
				Stem/Inverted										
No.	Location	Region	Loop	repeat	*J. regia*	‘Bokexiang’	*J. sigillata*	*J. hopeiensis*	*J. cathayensis*	*J. mandshurica*	*J. hindsii*	*J. major*	*J. nigra*	*J. cinerea*
1	*trnK-rps16*	LSC	3	9	–	–	–	Inverted	Inverted	Inverted	Inverted	Inverted	Inverted	Inverted
2	*trnS-trnG*	LSC	18	8	–	–	–	–	–	–	–	Inverted	–	–
3	*trnD-trnY*	LSC	3	9	–	–	–	Inverted	Inverted	Inverted	Inverted	Inverted	Inverted	Inverted
4	*trnE-trnT*	LSC	4	9	–	–	–	–	–	–	Inverted	Inverted	Inverted	Inverted
5	*trnT-psbD*	LSC	6	13	–	–	–	Inverted	Inverted	Inverted	–	–	–	–
6	*psbC-trnS*	LSC	7	11	–	–	–	–	Inverted	Inverted	Inverted	Inverted	Inverted	Inverted
7	*psaA-ycf3*	LSC	2	8	–	–	–	Inverted	Inverted	Inverted	–	–	–	–
8	*trnM-atpE*	LSC	7	13	–	–	–	–	–	–	–	–	Inverted	–
9	*petA-psbJ*	LSC	3	11	–	–	–	Inverted	Inverted	Inverted	–	–	–	–
10	*rrn4.5-rrn5*	IR	31	18	–	–	–	–	–	–	Inverted	–	–	–
11	*trnR-trnN*	IR	4	13	–	–	–	–	Inverted	–	Inverted	–	–	–
12	*ycf1*	SSC	6	13	–	–	–	Inverted	–	Inverted	–	–	–	–


**Juglans regia cp genome sequence was taken as standard reference during investigation on the inversion positions. Inversion events are determined for each of the other taxa by comparing with the cp genome sequence of *J. regia*. ‘–’ indicates no variation. The investigation results are highlighted with different colors for each section of genus Juglans.**

Each of the small inversions from *trnK-rps16* or *trnD-trnY* only occurred in section *Juglans*. The two small inversions from *trnT-trnD*, *psbA-ycf3* and *petA-psbJ* only occurred in section *Cardiocaryon*. The small inversion from *trnE-trnT* occurred within section *Rhysocaryon* and section *Trachycaryon* (including a single species *J. cinerea*). Each small inversion from *trnS-trnG*, *trnM-atpE*, or *rrn4.5-rrn5* occurred only in certain taxon within sect. *Rhysocaryon*. The small inversion from *trnE-trnT* was only observed in both section *Juglans* and section *Cardiocaryon*.

The small inversion from *psbC-trnS* occurred in section *Juglans*, including *J. hopeiensis* which is regarded as a natural hybrid between *J. mandshurica* and *J. regia* (molecular evidences from our experiments will be published in another article in detail). The small inversion in *ycf1* occurred in either *J. mandshurica* or *J. hopeiensis*. The 4 bp small inversion of *trnR-trnN* occurred simultaneously in part of taxa in section *Cardiocaryon* and section *Rhysocaryon*, showing no phylogenetic implication (**Table 4**).

### Phylogenetic Analysis

**FIGURE 3:**
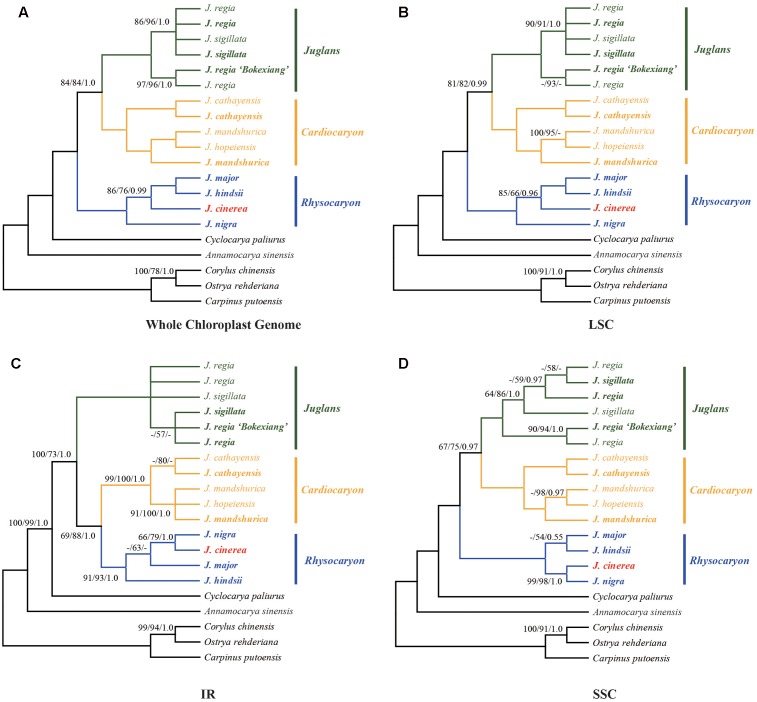
Phylogenetic relationships of *Juglans* inferred from maximum parsimony (MP), Bayesian inference (BI), and maximum likelihood (ML) analyses of different chloroplast genome data partitions. **(A)** Whole chloroplast genome. **(B)** LSC region. **(C)** IR region. **(D)** SSC region. Numbers near nodes indicate the MP bootstrap values (left) for each clade present in the 50% majority-rule consensus, ML bootstrap values (middle), and Bayesian posterior probability (right). Both MP and ML bootstrap support values = 100 and Bayesian posterior probability = 1.0 are not given at the nodes.

Phylogenetic analysis was conducted using each of the four sequence data sets: the complete chloroplast genome, LSC, SSC, or IR regions (**Figure 3**). The chloroplast genome sequences of *Corylus chinensis* (GenBank accession No. KX814336, Betulaceae), *Ostrya rehderiana* (GenBank accession No. KT454094, Betulaceae), *Carpinus putoensis* (GenBank accession No. KX695124, Betulaceae), *Cyclocarya paliurus* (GenBank accession No. KY246947, Juglandaceae), and *Annamocarya sinensis* (GenBank accession No. KX703001, Juglandaceae) were used as outgroups (**Figure 3**).

**FIGURE 4:**
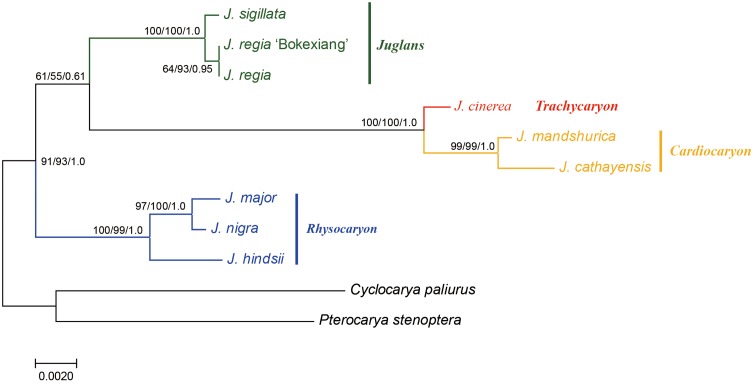
Phylogeny of *Juglans* based on ITS and ubiquitin ligase gene. Numbers near nodes indicate the MP bootstrap values (left) for each clade present in the 50% majority-rule consensus, ML bootstrap values (middle), and Bayesian posterior probability (right).

A combined sequence data set of a 724 bp length ITS sequence alignment with a 753 bp length ubiquitin ligase gene sequence alignment was used for the phylogenetic analyses. A total of 1,477 bp length nuclear DNA sequence alignment was used (**Figure 4**).

**FIGURE 5:**
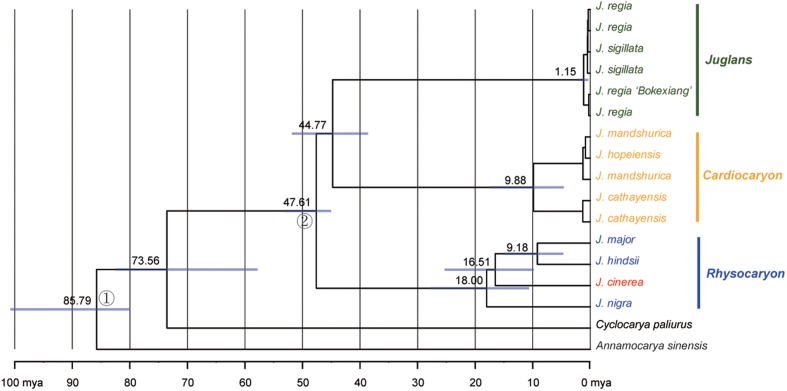
Chronogram obtained for *Juglans* under a Bayesian relaxed-clock approach based on the chloroplast genome dataset. Gray boxes indicate 95% confidence intervals on nodal ages.

The sequence data set from either the whole chloroplast genome or SSC region provided the best and almost identical resolution in the phylogenetic analyses with high bootstrap support value in comparison with the sequence data set from each of the rest two chloroplast regions (LSC and IR regions). Generally, the walnut taxa could be separated into three branches by the chloroplast DNA sequence data sets: (1) section *Juglans*, (2) section *Cardiocaryon*, and (3) section *Rhysocaryon* including *J. cinerea* which is closer to *J. nigra* (**Figure 3**). However, the combined sequence data set from the two nuclear DNA regions revealed a different phylogenetic topology of three branches in *Juglans*: (1) section *Juglans*, (2) section *Cardiocaryon* plus section *Trachycaryon* (*J. cinerea*) which is closer to *J. mandshurica*, and (3) section *Rhysocaryon* (**Figure 4**). *J. hopeiensis* which was classified in section *Cardiocaryon* showed a closer relationship with *J. mandshurica*. Based on the complete chloroplast genome sequence data, the divergence time between section *Juglans* and section *Cardiocaryon* was 44.77 Mya. The divergence time of section *Rhysocaryon* from other sections in the genus *Juglans* was 47.61 Mya (**Figure 5**).

## Discussion

### Structure, Size and Phylogenetic Information of Small Inversions

Large inversions are well characterized in the chloroplast genomes of various plant families/genera and the sequence data have been used to determine angiosperm lineages from the genus to phylum level (Jansen and Palmer, 1987; Milligan et al., 1989; Raubeson and Jansen, 1992; Hupfer et al., 2000; Kim and Lee, 2005). In contrast, limited plant groups were studied involving small inversions. For examples, a four base inversion associated with a hairpin secondary structure occurs within the *rpl*16 intron of the chloroplast genomes of some members of the genus *Chusquea* and related bamboo species (Poaceae) (Kelchner and Wendel, 1996). Sixteen small inversions ranging from 5 to 50 bp occurred in chloroplast genomes of phylogenetically distantly related groups of land plants including Poaceae, Fabaceae, and Solanaceae (Kim and Lee, 2005).

Within a single genus, Kim and Lee (2005) selected nine species of *Jasminum* (Oleaceae) to document the occurrence of the small inversions in closely related species. A 11 bp small inversion associated with a 19 bp inverted repeat was uncovered within the *trnL-F* non-coding regions in the chloroplast genome of *Jasminum* (Kim and Lee, 2005). In this study, 12 small inversions (loops) ranging from 2 to 31 bp in length were detected in chloroplast genomes of *Juglans* (Juglandaceae), they were associated with the inverted repeating sequences ranging from 8 to 18 bp in length. This indicated that there are large differences in the occurrence and distribution of small inversion between plant families. Our study further verified that small inversions are valuable genetic source for phylogenetic researches within a single genus, because more than 83.3% of them were found to be phylogenetically informative in revealing the genetic variations of *Juglans* plants at section and taxa levels.

In this study, a 4 bp small inversion in *trnR-trnN* intergenic spacer has no phylogenetic significance, indicating that careful analysis is necessary before use of small inversions.

### Phylogeny of *Juglans*

In this study, we explored and analyzed new genetic information by sequencing the complete chloroplast genomes and two nuclear DNA regions (ITS, and ubiquitin ligase gene) of nine *Juglans* taxa representing the four sections of the genus *Juglans* previously published based on morphological characteristics. The phylogenetic analyses were conducted using three algorithms, MP, ML, and BI methods. No significant difference was found among the algorithms.

According to analysis based on DNA sequences from two nuclear gene regions, the monotypic section *Trachycaryon* (*J. cinerea*) is a sister to the section *Cardiocaryon*. These two sections (*Trachycaryon* and *Cardiocaryon*) together further formed one branch which is a sister group to section *Juglans* or section *Rhysocaryon.* Sectional level divergence occurred in *Juglans* around 44.77 to 47.61 Mya in Eocene. These are similar to the previous reports (Aradhya et al., 2007).

The phylogenetic topology obtained using molecular data is generally identical with the tree topology obtained using morphological data, except for the placement of *J. cinerea* (Manning, 1978). In *Juglans*, the phylogenetic position of *J. cinerea* has been controversial (Aradhya et al., 2007; Laricchia et al., 2015). In this study, phylogenetic analysis based on the chloroplast genome sequences showed that *J. cinerea* was positioned within section *Rhysocaryon.* The closer relationship between *J. cinerea* and *J. nigra* is identical with their current geographical occurrence. The chloroplast genome data do not support the isolated position of the monotypic section *Trachycaryon* (*J. cinerea*) based on morphological characters.

The distributional ranges of the Tertiary fossils of butternuts (*J. cinerea*) and black walnuts (*J. nigra*) do not overlap except in the northwestern parts of the United States around 40° N latitude, strongly suggesting that they may have evolved independently as suggested by Hills et al. (1974).

Sequences from eight different regions of the chloroplast genome in 197 trees in *J. cinerea* sampled from their distribution area revealed 10 haplotypes (Laricchia et al., 2015). The phylogenetic incongruence for *J. cinerea* based on nuclear DNA sequences and/or chloroplast genome sequences might be potentially caused by hybridization. *Juglans* section *Trachycaryon* based on morphological characteristics was supported by neither nuclear nor chloroplast DNA sequences. This is still a mystery at current stage and will be a key point for us to challenge in future phylogenetic studies of the genus *Juglans* (Aradhya et al., 2007). Further study by sampling more individual trees/populations of *J. cinerea*, members from section *Rhysocaryon* and section *Cardiocaryon* and utilizing potential information from the whole nuclear genome sequence of *J. regia* (Martinez-Garcia et al., 2016) will be meaningful.

*Juglans hopeiensis* was shown maternally belonging to the same chloroplast lineage with *J. mandshurica* in section *Cardiocaryon* in this study. This result is identical with the previous studies which suggested that *J. hopeiensis* is an inter-specific hybrid between *J. mandshurica* and *J. regia* (Rehder, 1940; Lu et al., 1999; Wu et al., 2000; Aradhya et al., 2007). Further study is necessary for a more clear elucidation involving the origin of *J. hopeiensis*.

## Conclusion

This study reports the comparative genomic analysis results of nine *Juglans* chloroplast genome sequences with detailed gene annotation. More than 83.3% of the small inversions in the chloroplast genomes provided valuable genetic information for phylogenetic researches at taxon and section levels in *Juglans*. All of the *Juglans* taxa were discriminated completely with high bootstrap support values. The molecular taxonomy of *Juglans* is almost compatible to the currently accepted morphological taxonomy except *J. cinerea* (section *Trachycaryon*). The existence of the monotypic section *Trachycaryon* (*J. cinerea*) based on morphological characteristics was supported by neither nuclear nor chloroplast DNA sequences. The systematic position of *J. cinerea* shifted from a member of *J.* section *Cardiocaryon* based on the combined nuclear DNA sequence data set to a member of the section *Rhysocaryon* based on the chloroplast genome sequence data set. Further studies centering *J. cinerea* by sampling more samples will be helpful for clarifying the phylogenetic placement of *J. cinerea*. Sectional level divergence time of *Juglans* was 44.77 to 47.61 Mya in Eocene. These results obtained in this study are valuable for future researches on global *Juglans* genetic diversity and will enhance our understanding of the phylogenetic evolution of the Juglandaceae.

## Author Contributions

WD conceived and designed the experiments, performed the experiments, conducted the chloroplast genome assembling, analyzed the data, wrote the paper, prepared figures, and/or tables, reviewed drafts of the paper. CX performed the experiments, analyzed the data, wrote the paper, reviewed drafts of the paper. WL, YiL, and XX conceived and designed the experiments, contributed reagents/materials/analysis tools, wrote the paper, reviewed drafts of the paper. YaL performed the experiments, prepared figures and/or tables. XJ wrote the paper, reviewed drafts of the paper. ZS conceived and designed the experiments, performed the experiments, analyzed the data, contributed reagents/materials/analysis tools, wrote the paper, reviewed drafts of the paper.

## Conflict of Interest Statement

The authors declare that the research was conducted in the absence of any commercial or financial relationships that could be construed as a potential conflict of interest.
