# Supplementary material for: Phylogenetic Resolution in Juglans Based on Complete Chloroplast Genomes and Nuclear DNA Sequences
Source: Front Plant Sci. 2017 Jun 30;8:1148. doi: 10.3389/fpls.2017.01148 (PMC5492656; doi:10.3389/fpls.2017.01148)
Supplement: Supplementary file 1 [file Table_1.DOCX]

**TABLE S1 | Summary of the chloroplast genome sequencing data for five *Juglans* species using Illumina HiSeq 4000.**

| **Species** | **Raw data no.** | **Mapped read no.** | **Mapped to reference genome (%)** | **cp gemome coverage (×)** |
| --- | --- | --- | --- | --- |
| *J. cathayensis* | 18,184,763 | 799,986 | 2.20% | 751 |
| *J. cinerea* | 29,818,482 | 1,160,474 | 1.95% | 1,086 |
| *J. hindsii* | 13,050,720 | 1,744,889 | 6.69% | 1,632 |
| *J. major* | 8,801,265 | 303,763 | 1.73% | 284 |
| *J. nigra* | 12,095,625 | 584,962 | 2.42% | 547 |
