# Supplementary material for: Phylogenetic Resolution in Juglans Based on Complete Chloroplast Genomes and Nuclear DNA Sequences
Source: Front Plant Sci. 2017 Jun 30;8:1148. doi: 10.3389/fpls.2017.01148 (PMC5492656; doi:10.3389/fpls.2017.01148)
Supplement: Supplementary file 2 [file Table_2.DOCX]

**TABLE S2 | A list of genes encoded in the chloroplast genome of *Juglans*.**

| **Category for genes** | **Group of gene** | **Name of gene** |
| --- | --- | --- |
| Photosynthesis related genes | Photosystem Ⅰ | *psaA, psaB, psaC, psaI, psaJ* |
|  | Photosystem Ⅱ | *psbA, psbB, psbC, psbD, psbE, psbF, psbH, psbI, psbJ, psbK, psbL, psbN, psbT, psbZ* |
|  | Cytochrome b/f compelx | *petA, *petB, *petD, petG, petL, petN* |
|  | ATP synthase | *atpA, atpB, atpE, *atpF, atpH, atpI* |
|  | Cytochrome c synthesis | *ccsA* |
|  | Assembly/stability of photosystem Ⅰ | **ycf3, ycf4* |
|  | NADPH dehydrogenase | **ndhA, *ndhB, ndhC, ndhD, ndhE, ndhF ,ndhG, ndhH, ndhI, ndhJ, ndhK* |
|  | Rubisco | *rbcL* |
| Transcription and translation related genes | Transcription | *rpoA, rpoB, *rpoC1, rpoC2* |
|  | Ribosomal proteins | *rps2, rps3, rps4, rps7, rps8, rps11, *rps12, rps14,rps15, *rps16, rps18, rps19, rpl2, rpl14, *rpl16, rpl20, rpl22, rpl23, rpl32, rpl33,rpl36* |
| RNA genes | Ribosomal RNA | *rrn5, rrn4.5, rrn16, rrn23* |
|  | Transfer RNA | **trnA-UGC, trnC-GCA, trnD-GUC, trnE-UUC, trnF-GAA,trnG-GCC, *trnG-UCC, trnH-GUG, trnI-CAU, *trnI-GAU, *trnK-UUU, trnL-CAA, *trnL-UAA, trnL-UAG, trnfM-CAU, trnM-CAU, trnN-GUU, trnP-UGG, trnQ-UUG,trnR-ACG, trnR-UCU, trnS-GCU, trnS-GGA, trnS-UGA, trnT-GGU, trnT-UGU, trnV-GAC, *trnV-UAC, trnW-CCA, trnY-GUA* |
| Other genes | RNA processing | *matK* |
|  | Carbon metabolism | *cemA* |
|  | Fatty acid synthesis | *accD* |
|  | Proteolysis | **clpP* |
| Genes of unknown function | Conserved reading frames | *ycf1, ycf2* |

Intron-containing genes are marked by asterisks (*).
